# Bicuspid Aortic Valve and Premature Ventricular Beats in Athletes

**DOI:** 10.3390/ijerph191912188

**Published:** 2022-09-26

**Authors:** Gloria Modica, Fabrizio Sollazzo, Massimiliano Bianco, Michela Cammarano, Riccardo Pella, Riccardo Monti, Vincenzo Palmieri, Paolo Zeppilli

**Affiliations:** Sports Medicine Unit, Fondazione Policlinico Universitario A. Gemelli IRCCS, Catholic University, 00168 Rome, Italy

**Keywords:** bicuspid aortic valve, premature ventricular beats, athletes, sport eligibility

## Abstract

Background: The aim of this study was to identify a possible link between bicuspid aortic valve (BAV) and premature ventricular beats (PVBs), particularly from left and right ventricular outflow tracts, and to investigate possible associations between these arrhythmias and echocardiographic abnormalities. Methods: A comparison of sportspeople with and without BAV was performed to identify PVBs’ occurrence in these two series. Then, subdividing the BAV group on the presence of cardiovascular complications due to BAV, we compared arrhythmic features between these two subgroups and echocardiographic findings between athletes with and without left and right outflow tract PVBs. Results: PVBs in 343 athletes with BAV were compared with 309 athletes without BAV, showing an increased frequency (29% vs. 11.8%, *p* < 0.001; OR 3.1; CI 2.1–4.7) and origin from the left (18.4% vs. 3.2%, *p* < 0.001, OR 6.7; CI 3.4–13.4) and right (15.2% vs. 3.6%, *p* < 0.001, OR 4.8; CI 2.5–9.5) outflow tracts compared to other ventricular areas (fascicular PVBs *p* = 0.81, other morphologies *p* = 0.58). No difference in PVBs’ occurrence was found between near normal valve BAV and pathological BAV, nor was a difference in echocardiographic characteristics found between patients with and without outflow tract arrhythmias. Conclusions: A possible causal link between BAV and PVBs was highlighted, but no association between PVBs and complicated BAV was emphasized.

## 1. Introduction

Bicuspid aortic valve (BAV) is one of the most frequent congenital heart diseases, with a prevalence of 0.5–2% (male to female ratio 3:1) in the general population [1].

BAV can be found as an isolated defect or associated with other abnormalities, such as aortic dilatation [2] and aortic coarctation [3]. BAV is also genetically related to left-sided lesions such as hypoplastic left heart syndrome [4], syndromes characterized by aortic dilatation (e.g., Marfan [5], Loeys–Dietz syndromes [6]) and obstructive left ventricular outflow tract (LVOT) defects (e.g., Turner [7], Shone’s [8], Williams’ syndromes [9]). Several genetic defects have been linked to this malformation [2,10], to the extent that consensus guidelines now recommend the screening of first-degree relatives [11].

Even though the pathogenetical basis of BAV remains to be fully elucidated, most evidence supports the current interpretation of BAV as a multifactorial disease, in which both genetic and hemodynamic factors seem to play a key role in explaining the heterogeneity of phenotypes and natural history [12].

Some authors have hypothesized a role for neural crest (NC) cells in the pathogenesis of BAV. In fact, from an embryological point of view, the neural crest (NC) plays an important role in the development of cardiac structures, in particular the outflow tract and the aortic arch [13,14], and also the conduction system [15,16]: several studies have demonstrated the presence of smooth muscular cells (SMCs) in these structures that derive from the NC, whereas the SMCs in the descending aorta derive from the paraxial mesoderm [17].

### BAV and Sports

Assessing the eligibility for competitive and/or professional sports of athletes with BAV can be challenging. In fact, all possible co-pathologies and complications must be researched and “weighed” in the overall assessment, also considering that the impact of regular training on the natural course of BAV is not fully established [18,19]. For this reason, current guidelines aim to highlight any structural and/or functional cardiac alterations in athletes with BAV, which may lead to disqualification from practising sport [19].

One of the less investigated aspects is the link between BAV, sport activity and the incidence of premature ventricular beats (PVBs), especially those originating from the left and right ventricular outflow tracts [20].

PVBs are a common ECG finding in the general population and are recorded in up to 75% of healthy individuals undergoing 24-h ambulatory ECG monitoring, with an age-related increase in prevalence [21,22] but no clear relationship with the degree of training [23,24].

Under the hypothesis that BAV is a malformation linked to an anomalous migration of the neural crest cells, which are involved in in the development of the outflow tracts, we sought to test the hypothesis that there is a correlation between this anomaly and arrhythmias originating from these cardiac areas, and that these arrhythmias are not related to a worsening of the patient’s overall clinical profile. For this reason, we tried to assess whether BAV is linked to PVBs from the left and right outflow tracts, and that these arrhythmias are not related to the morphological and functional status of the aortic valve, aortic root and left ventricle.

## 2. Materials and Methods

We retrospectively analysed the database of Sport Medicine Unit, Fondazione Policlinico Universitario A. Gemelli IRCCS, between 1987 and 2020, in order to extract the records of sportspeople (competitive and non-competitive) with BAV (BAV), discovered incidentally during pre-participation screening or who came to our centre for counselling after diagnosis. We compared these subjects with a group of athletes without BAV, matched for age and sex (NOT-BAV). We defined competitive athletes according to the 16th Bethesda conference, which defines a “competitive athlete” as an individual who participates in an organized team or in a sport that requires regular competition against others, places a high premium on excellence and achievement and requires some form of systematic and intense training [25]. Furthermore, “non-competitive athletes” refers to individuals engaged in recreational or leisure-time sports activities, either on a regular basis or intermittently [26].

The inclusion criteria were:

1. Practicing sports on a non-competitive, competitive or professional level.
2. Having undergone a 12-lead resting electrocardiogram (ECG), stress-test ECG and 2-dimensional trans-thoracic echocardiography (ECHO).
3. No cardiovascular symptoms, known cardiovascular disease (other than BAV) and ECG abnormalities except for the possible presence of PVBs.
4. As for the control group, no ECHO evidence of cardiovascular anomalies, i.e., normal chamber size; normal volume and wall thickness; normal left ventricular systolic and diastolic function; absence of intracardiac shunts; normal valvular function and morphology; absence of pericardial effusion; normal size of ascending aorta and arch with normal flow velocities.
5. No history of cardiac surgery.

In both the BAV and the NOT-BAV group, we took into account any athlete who showed at least one PVB during ECG or stress-test ECG. We classified ventricular arrhythmias, based on current criteria [27], as follows:

– Morphology: LVOT origin for PVBs with left bundle branch block pattern, negative complex in V1, precordial S/R transition before V3 and inferior QRS axis in the limb leads, suggestive of the origin from the left ventricular outflow tract; right ventricular outflow tract (RVOT) origin for PVBs with negative QRS complex in V1, precordial S/R transition beyond V4 and inferior QRS axis in the limb leads; fascicular origin for PVBs with right bundle branch block pattern, superior or inferior axis in the limb leads and QRS ≤ 120 msec; other for all other morphologies.
– Number of morphologies: monomorphic if only one morphology was found per patient; polymorphic if more than one morphology was present per patient.
– Complexity: complex if PVB couplets and/or triplets and/or ventricular tachycardia (VT) were present.
– Relationship with exertion: exercise-related PVBs if present only during the exercise phase of the stress-test ECG, not exercise-related PVBs if present at rest and/or during the recovery phase of the stress-test ECG.

Stress-test ECG was performed using a cycle ergometer and incremental protocol of 30 watt/2 min until muscle exhaustion or the criteria defined by international guidelines for discontinuation were reached [28], with continuous monitoring of ECG during effort phase and for 10 min during recovery. Firstly, we compared the occurrence of PVBs, in particular PVBs with outflow tracts’ pattern between sportsmen with and without BAV. Secondly, by subdividing the population with BAV into “near normal valve” (NNV) [29] (without any possible cardiovascular complications related to BAV, i.e., no significant aortic stenosis, no more than trivial regurgitation, normal aortic dimensions and no associated cardiovascular anomalies such as aortic coarctation or mitral valve prolapse) and “pathological BAV” (PBAV), which includes all other cases, we sought to highlight whether there were any differences in ventricular arrhythmias between these two series.

Finally, we tested the hypothesis that BAV athletes with PVBs from left and right outflow tracts had no worse echocardiographic status than athletes without this sort of ventricular arrhythmias.

### Statistical Analysis

The normal distribution of all continuous variables was examined using the Shapiro–Wilk test, and data are presented as mean ± SD or median (IQR) accordingly. Categorical variables were reported as absolute numbers and percentages.

Comparisons were performed using chi-squared or Fisher exact tests in case of categorical variables, whereas Mann–Whitney U-test was used to check differences in case of continuous variables.

Differences were considered significant with a *p* < 0.05. Statistical analysis was carried out with SPSS 26.0.

## 3. Results

We found 343 records of sportsmen with BAV meeting the inclusion criteria (313 males, 91.3%), of which 186 (54.2%) were competitive and 157 (45.8%) non-competitive athletes.

The NOT-BAV group was composed of 309 sportsmen (270 males, 87.4%), of which 208 (67.3%) were competitive and 101 (32.7%) non-competitive. The two populations were homogeneous for age and gender (Table 1).

We found a 29% occurrence (100/343 people) of PVBs in the BAV group vs. 11.8% (36/309 people) in the NOT-BAV group (*p* < 0.001; OR 3.1; CI 2.1–4.7).

As regard the relationship with the exertion, in the BAV group a higher occurrence of exercise-related PVBs in respect to not exercise-related PVBs was not noted (*p* = 0.624, CI 0.8–1.3); instead, comparing the BAV and NOT-BAV groups, as expected, BAV athletes showed more frequently both exercise-related (19.8% vs. 8.4%, *p* < 0.001, OR 2.7; CI 1.7–4.4) and not exercise-related PVBs (18% vs. 5.5%; OR 3.8; CI 2.2–6.6).

In the BAV group, the PVBs’ morphology was LVOT in 63 cases out of 343 (18.4%), RVOT in 52 (15.2%), LVOT and RVOT (polymorphic) in 15 (4.4%), fascicular in 4 (1.2%) and other in 20 (5.8%). In the control group, the morphology was LVOT in 10 cases out of 309 (3.2%), RVOT in 11 (3.6%), LVOT and RVOT in 2 (0.6%), fascicular in 3 (1.0%) and other in 1 (4.9%). A statistically significant difference between BAV and NOT-BAV was present as regard the rate of occurrence of RVOT and LVOT morphologies, whereas no significant difference was found for fascicular PVBs and other morphologies.

Among the BAV, 24 subjects out of 343 (7.0%) had complex PVBs, whereas in the NOT-BAV they were present in 4 cases out of 309 (1.2%, *p* < 0.001; OR 5.7, CI 2.0–16.7); these complex PVBs in the BAV group were monomorphic in all cases, and they arose from outflow tracts in most of the cases (20 out of 24, 83%).

As for the number of morphologies, polymorphic arrhythmias were found in 18 (5.2%) BAV athletes, whereas among NOT-BAV athletes they were found in 10 (3.2%) people (*p* = 0.21, OR 1.7, CI 0.8–3.6). (Table 2).

When comparing non-competitive and competitive sportspeople with regard to the presence of PVBs within both the BAV group and the NOT-BAV group, no significant differences were found (33.3% vs. 37% in BAV, *p* = 0.8; OR 1.2; CI 0.3–4.1; 12.7% vs. 7.2% in NOT-BAV, *p* = 0.207; OR 0.53; CI 0.9–1.4).

Within the group with BAV, the echocardiography allowed the participants to be classified into two groups (NNV and PBAV). According to this subdivision, the NNV group consisted of 99 people, while the PBAV numbered 244. When comparing the arrhythmias (morphology, complexity, number of morphologies, relationship with exertion) between these two series, no difference was noted; specifically, there was no different occurrence of PVBs from LVOT and/or RVOT between the two series (Table 3).

To confirm the absence of a relationship between the occurrence of ventricular outflow tract arrhythmias and echocardiographic abnormalities, we finally tried to compare the echocardiographic parameters between people with and without these type of PVBs (Table 4): our analysis did not point out a worse echocardiographic status for athletes with PVBs from outflow tracts.

## 4. Discussion

Bicuspid aortic valve (BAV) is a congenital valvular heart disease with a natural history that sometimes evolves into complications (e.g., aortic dilatation, valvular stenosis or regurgitation and, rarely, aortic dissection); hence, the focus of follow-up is on echocardiography in order to identify cases that should be addressed by surgical correction [19,30]. As intense sport practice has a deep impact on both cardiovascular morphology and haemodynamics, its role in the development of BAV complications still needs to be fully elucidated [18,19]. Ventricular arrhythmias are often signs of haemodynamic deterioration and can be the cause of major events such as sudden cardiac death, the main concern of sport cardiologists [24]. At the same time, the occurrence of ventricular arrhythmias is very common in sportspeople, and represents one of the most important reasons to perform further investigations in order to properly stratify individual risk [31]. Whether BAV may have an impact on the occurrence of PVBs in athletes is an aspect that has been poorly studied: only one study to date has considered the possible clinical implications of the presence of ventricular arrhythmias in BAV, but a greater frequency of PVBs was not found in the population of athletes with BAV than in a healthy control group [20].

Our study, for the first time, showed an increased frequency of ventricular arrhythmias both at rest and during exercise in a group of sportspeople with BAV in comparison to a control group with normal hearts. As expected, the level of sports practice (competitive or non-competitive) does not seem to affect the occurrence of PVBs.

Moreover, these arrhythmias seem to originate mainly from the right and left outflow tracts, which embryologically derive from neural crest cells, the same cells that originate the cardiac conduction system: in fact, no difference in the occurrence of PVBs between the two groups was found for PVBs with fascicular morphology or with morphologies other than LVOT or RVOT. According to recent work by Corrado et al., PVBs from outflow tracts are defined as having a “common” morphology, as they are often found in athletes, typically in association with normal cardiac morphology and function and generally without an increased risk of sport-related sudden cardiac death [29]. Therefore, finding an association between BAV and ventricular arrhythmias and stratifying the risk of PVBs is extremely important when assessing athletes with this condition. From this point of view, we investigated the relationship between arrhythmias and echocardiographic characteristics of patients, firstly by subdividing the population on the basis of the development of typical complications of BAV. In doing so, we noticed that there is no difference in the frequency and morphology of and the relationship with exertion for PVBs between athletes with a NNV BAV and those with a PBAV. According to these data, it is reasonable to assume that, as a general rule, the detection of PVBs in athletes with a BAV is not typically associated with a worse echocardiographic status. A possible practical consequence of this finding is that the detection of ventricular arrhythmias in an athlete with BAV is unlikely to suggest a worse valve condition in the first instance. Moreover, as most PVBs in athletes with BAV appear to arise from both outflow tracts, the absence of a difference in echocardiographic parameters in athletes with BAV between people with and without outflow tract arrhythmias reinforces the idea that PVBs from outflow tracts are a typical feature of BAV, not having a relationship with the individual’s cardiovascular status and, generally speaking, not increasing the risk of adverse events. Following this interpretation, we are prone to consider an increased presence of automatic foci in the outflow tract, linked to abnormal migration of the neural crest cells in the BAV [14], as more likely than the possible development of re-entry circuits due to disease-related haemodynamic factors [12] as an explanation for the increased presence of outflow tract PVB in athletes with BAV.

In light of our results, we believe that the identification of outflow tract arrhythmias should also be considered a “common” finding in BAV, which should prompt careful evaluation, but which could be considered “favourable”, until proven otherwise, as in apparently “healthy” athletes.

In this regard, we would like to recall that, nowadays, the Italian guidelines on competitive sport eligibility recommend an ECHO, a stress-test ECG and a 24-h Holter ECG, possibly including a training session to evaluate competitive athletes with BAV [32], while other international guidelines on the same topic (European Society of Cardiology [19], American Heart Association [33]) suggest additional examinations beyond the ECHO only in the presence of aortic dilatation.

### Study Limitations

Firstly, as our population consisted exclusively of athletes with BAV, our considerations are limited to them and cannot be generalised (in particular to people with advanced clinical status and potential indication for surgical correction).

Our series does not allow us to provide indications on the role of PVBs on the natural history of bicuspid aortic valve, as we have not carried out follow-up analysis (which are in progress): thus, we limited ourselves to highlighting the possible clinical implications of the observed relationship between BAV and PVBs.

Furthermore, we do not have any further data on 24-h ambulatory ECG monitoring, which could add different information about PVBs (i.e., frequency over 24 h) and on other diagnostic examinations, in particular imaging studies (i.e., Cardiac Magnetic Resonance and Contrast-enhanced Coronary Computed Tomography Angiography), electrophysiological studies and/or genetic screening, which could allow us to evaluate the presence of any kind of pathological issue.

Lastly, we are also aware of the possibility of the misinterpretation of PVBs’ morphology, commonly due to the variable spread of excitation through the ventricle (in particular in patients with coronary artery disease), the presence of different degrees of fusion with the subsequent sinus complex and additional aberrant conduction secondary to the short RR interval in the second beat of a couplet [34].

## 5. Conclusions

BAV is very common in the general population, with a possible risk of serious complications. To date, there is no certainty that intensive sports practice does not increase the risk of these complications in the long term. Therefore, competitive sports practice for athletes with BAV requires extensive evaluation and proper follow-up over time. The relationship between PVBs and BAV has been scarcely explored in recent years. Our series demonstrated that PVBs are more frequent in athletes with BAV than in the general population and that they mainly originate from the left ventricular and right ventricular outflow tracts. Moreover, no difference was found in the occurrence of PVBs between people with near normal and pathological BAV. Lastly, echocardiographic findings were similar by dividing the BAV group into people with and without outflow tract arrhythmias. Our study, for the first time, highlights a possible causal link between BAV and PVBs from the outflow tracts and points out no association between the presence of PVBs and complicated BAV.

## Figures and Tables

**Table 1:** Homogeneity test for the main groups’ parameters.

Parameters	BAV (343)	NOT-BAV (309)	*p*-Value
Gender (M/F)	313/30	270/39	0.126
Age (years)	21 (IQR 15; 22)	20 (IQR 16; 27)	0.646
Weight (kg)	70 (IQR 56; 79)	72 (IQR 70; 78.1)	0.007
Height (cm)	173 (IQR 165; 180)	176 (IQR 168.8; 182)	<0.001
Sport level (competitive/non-competitive)	186/157	208/101	<0.001

**Table 2:** PVBs’ rate of occurrence, relationship with exertion, morphology, number of morphologies and complexity in BAV group vs. controls.

PVBs (Number of Athletes) ^A^	BAV N = 343 (%)	Controls (N = 309) (%)	*p*-Value	Odds Ratio; Confidence Interval
Occurrence	100 (29%)	36 (11.8%)	<0.001 *	3.1; 2.1–4.7
Exercise-related	68 (19.8%)	26 (8.4%)	<0.001 *	2.6; 1.6–4.1
Not exercise-related	62 (18%)	17 (5.5%)	<0.001 *	4.2; 0.8–1.9
Exercise-related vs. not exercise-related ^B^	68/275 vs. 62/281 ^B^	/	0.624	1.1; 0.8–1.3
*PVBs morphologies*				
LVOT	42 (12.2%)	10 (3.2%)	<0.001 *	4.2; 2.1–8.5
RVOT	52 (15.2%)	11 (3.6%)	<0.001 *	4.8; 2.5–9.5
Fascicular	4 (1.2%)	3 (1.0%)	0.81	1.2; 0.3–5.4
Other	20 (5.8%)	15 (4.9%)	0.58	1.2; 0.6–2.4
Polymorphic ^C^	18 (5.2%)	10 (3.2%)	0.21	1.7; 0.8–3.6
Complex	24 (7%)	4 (1.3%)	<0.001	5.7; 2.0–16.7

PVBs = premature ventricular beats; LVOT = left ventricular outflow tract; RVOT = right ventricular outflow tract; * = statistically significant difference. ^A^ Athletes may show both exercise and not exercise-related arrhythmias, and more than one morphology of PVBs; ^B^ exercise-related PVBs/BAV exercise-related PVBs; not exercise-related PVBs/BAV not exercise-related PVBs; ^C^ among them, 15 athletes had both LVOT and RVOT morphologies.

**Table 3:** PVBs’ rate of occurrence, relationship with exertion, morphology, number of morphologies and complexity comparison between near normal valve BAV and pathological valve BAV.

PVBs (Number of Athletes)	Near Normal Valve (N = 99)	Pathological Valve (N = 244)	*p*-Value
Occurrence	32 (32.3%)	68 (27.9%)	0.41
Exercise-related	20 (20.2%)	48 (19.7%)	0.91
Not exercise-related	20 (20.2%)	42 (17.2%)	0.52
LVOT	15 (15.2%)	27 (11.1%)	0.30
RVOT	15 (15.2%)	37 (15.2%)	1.00
Outflow tracts	34 (34.3%)	66 (27%)	0.18
Fascicular	0 (0%)	4 (1.6%)	0.20
Other	8 (8.1%)	12 (4.9%)	0.26
Polymorphic	6 (6.1%)	12 (4.9%)	0.67
Complex	6 (6.1%)	18 (7.4%)	0.67

PVBs = premature ventricular beats; LVOT = left ventricular outflow tract; RVOT = right ventricular outflow tract.

**Table 4:** Comparison of echocardiographic parameters between patients with and without occurrence of outflow tract arrhythmias.

Echo Parameters	Outflow Tracts Arrhythmias (N = 79)		No Outflow Tracts Arrhythmias (N = 264)		*p*-Value
BAV anatomy (antero-posterior/latero-lateral)	64	15	210	54	0.78
Left ventricular ejection fraction	61.1 ± 4.8		61.6 ± 4.8		0.28
Mitral E/A ratio	1.85 ± 0.64		1.94 ± 0.60		0.24
Aortic root dilatation	34 (43%)		133 (50.4%)		0.25
Left ventricular dilatation	11 (13.9%)		48 (18.2%)		0.38
Left ventricular hypertrophy	7 (8.9%)		34 (12.9%)		0.33
More than trivial aortic regurgitation	21 (26.6%)		81 (30.7%)		0.48
Significant aortic obstructive gradient	4 (5.1%)		21 (8.0%)		0.39
Associated heart diseases	18 (22.8%)		63 (23.9%)		0.84

## Data Availability

The data presented in this study are openly available on the studies referenced in the figures, and individual data of each can be consulted in the original manuscripts.
